# The acceptability to women of techniques for managing an impacted fetal head at caesarean section and of randomised trials evaluating those techniques: a qualitative study

**DOI:** 10.1186/s12884-021-03577-z

**Published:** 2021-02-02

**Authors:** Gabriella Romano, Eleanor Mitchell, Rachel Plachcinski, Natalie Wakefield, Kate Walker, Susan Ayers

**Affiliations:** 1 Centre for Maternal and Child Health Research, School of Health Sciences, City, University of London, Northampton Square, London, EC1V 0HB UK; 2 Nottingham Clinical Trials Unit, University of Nottingham, Building 42 University Park, Nottingham, NG7 2RD UK; 3 National Childbirth Trust, NCT, 30 Euston Square, London, NW1 2FB UK; 4 Division of Child Health, Obstetrics and Gynaecology, School of Medicine, Lenton, University of Nottingham, Nottingham, NG7 2RD UK

**Keywords:** Feasibility, Acceptability, Caesarean, Techniques for impacted fetal head, Qualitative

## Abstract

**Background:**

This study aimed to explore women’s views on the acceptability of different techniques for managing an impacted fetal head at caesarean; and the feasibility and acceptability of conducting a trial in this area*.*

**Methods:**

Qualitative semi-structured interviews with a systematic sample of women who experienced second stage emergency caesarean section at a tertiary National Health Service (NHS) hospital in England, UK. Thematic analysis was used to extract women’s views.

**Results:**

Women varied in their perceptions of the acceptability of different techniques for managing impacted fetal head. Trust in medical expertise and prioritising the safety of the baby were important contextual factors. Greater consensus was found around informed choice in trials where subthemes considered the timing of invitation, reduced capacity to give consent in emergency situations, and the importance of birth outcomes and having good rapport with healthcare professionals who invite women into trials. Finally, women reflected on the importance of supportive antenatal and postpartum education for impacted fetal head.

**Conclusions:**

This research provides information on the acceptability of techniques and any trial to evaluate these techniques. Findings illustrate the importance of context and quality of care to both acceptability and approaching women to take part in a future trial.

**Supplementary Information:**

The online version contains supplementary material available at 10.1186/s12884-021-03577-z.

## Background

Emergency caesarean section (CS) accounts for 16% of all births in the UK [1] of which at least 5% are done at full dilatation in the second stage of labour, that is 4830 births per annum [2]. Emergency CS performed in the second stage of labour has greater perinatal and maternal morbidity than those performed in the first stage [3]. Second stage CS may be complicated by the fetal head being deeply impacted in the maternal pelvis which occurs in 1.5% of all emergency CS [3, 4]. As an obstetric emergency, the challenge for the clinical team is to disengage the head by hand due to minimal space between the bony maternal pelvis and the deeply impacted fetal head. Complications can arise for mother and baby and include: major haemorrhage secondary to uterine or vaginal tears, longer hospital stay and delivery times, greater risk of bladder trauma, injury to the baby [3] such as bony fractures, hypoxic brain injury or occasionally death.

There is currently no UK or international guidance on which techniques to employ to manage an impacted fetal head at CS. Numerous techniques to assist in delivery of an impacted head are reported but the superiority of one technique over another is contentious [5]. Most commonly used techniques include: the reverse breech or ‘pull technique’, the ‘push technique’, and fetal pillow [6]. A review of randomised controlled trials (RCTs) of different techniques concluded there is insufficient evidence to support or refute the use of any technique to facilitate infant birth during a difficult caesarean section [5]. In the UK, obstetricians and midwives do not receive standardised training. Thus, there is no consensus on best practice and research is needed to inform the methods used by clinical teams during these critical incidents and determine which is the most effective.

The objective of this research was to qualitatively examine women’s views on the acceptability of different techniques for managing an impacted fetal head during emergency CS; and the feasibility and acceptability of conducting a RCT in this area. The Medical Research Council framework [7] provides guidance on the development and evaluation of complex interventions [8]. A range of methods can be used to examine feasibility, with qualitative studies increasingly incorporated prior to or during RCTs to assess acceptability and feasibility. This qualitative study is part of a larger programme of work, including national surveys of current practice and parents’ views, and a Delphi study to agree which techniques should be tested. This will provide valuable information needed to determine whether it is possible to conduct an RCT in this area.

## Methods

### Sample and recruitment

A systematic sample of women was recruited through one hospital in England. Women were eligible if they had experienced a second stage CS in the 24 months prior to recruitment; were aged 16 years or older; had adequate spoken English; and were able to give informed consent. There were no exclusion criteria. All women who were eligible over a 24-month period (*n* = 140) were identified from medical records. Of these, 80 were invited to participate: 50 who lived in deprived areas (i.e. an Index of Multiple Deprivation Decile (IMD) of 1 or 2) and 30 in less deprived areas (IMD of 3–10). Women who were interested in taking part returned a pre-paid letter to the research team providing contact details and a signed consent form. Postal responses were returned by 19 women interested in taking part: 17 consented and 2 expressed interest but did not return the consent form. Of women who consented, 9 (53%) were available to be interviewed. The remaning 8 women were not available to be interviewed during the study period.

Women were offered an initial telephone call with a research psychologist so they could ask questions and state their preference for a one-to-one interview or focus group. Nearly all women preferred one-to-one interviews. Interviews were conducted at the university campus by two practitioners: a psychologist experienced in qualitative research with vulnerable groups, and a National Childbirth Trust practitioner experienced in explaining birth-related information in an accessible way. Women were able to bring their baby to the interview and were reimbursed for travel and childcare costs.

Before the interview participants provided sociodemographic information and were given brief information about the study and the different techniques used for impacted fetal head. Participants were shown photos and physical prototypes of the fetal pillow and Tydeman tube instruments during the presentation. A 45-min semi-structured interview was then conducted to explore participants’ experiences and views on the acceptability of different techniques and a RCT of techniques using a topic guide. This interview guide was developed for this study and is provided as an Appendix (S1). Not all women were aware of whether or not they had experienced an impacted head. Women were shown photographs and diagrams to illustrate the different techniques and where appropriate were shown a real life version (fetal pillow; Tydeman Tube) that they could handle themselves. A model pelvis and baby were also used to explain the different techniques.

If women wanted additional information about their birth and/or referral to an obstetrician to find out more they were encouraged to contact their GP. Information was given in the Participant Information Sheet for recommendations of who they could contact. The number of interviews conducted was dependent on women’s availability. All interviews were audio recorded and transcribed using an external transcription agency.

### Data analysis

Audio recordings were transcribed verbatim and analysed using systematic thematic analysis [9]. A combined inductive and deductive approach was used with the following steps: first, all transcripts were read to become familiar with the data. Transcripts were then re-read and initial codes identified and coded. When no further codes emerged (i.e. data saturation) all the codes were examined by two researchers (GR and SA) to agree which were most frequent or could be combined into key themes. Themes were cross-checked against coded quotes to ensure reliability of coding and that main themes were represented. Analysis was conducted using NVivo12 software [10].

## Results

### Sample characteristics

Participants were 30–40 years of age and all White British. Five participants were married and the remainder (*n* = 4) were living with their partner. The majority of women were educated to degree level (*n* = 5) or above (*n* = 1). All participants were in employment across a range of industries including healthcare (*n* = 3), retail (*n =* 3), probation (*n* = 1), education (*n =* 1) and catering (*n =* 1).

### Main themes

Three main themes were identified: (1) *Acceptability of different techniques;* (2) *Informed choice in trials;* and (3) *Birth education*. Each theme had a number of subthemes as shown in Table 1.

**Table 1:** Themes and subthemes

Themes	Subthemes	Participants N (%)
Acceptability of different techniques	Level of invasiveness	5 (55%)
	Security in practitioner expertise	5 (55%)
	Baby safety	4 (44%)
Informed choice in trials	Timing of invitation	7 (77%)
	Capacity to make an informed choice	5 (55%)
	Birth outcome	3 (33%)
	Importance of rapport	4 (44%)
Birth education	Antenatal education	9 (100%)
	Postpartum information	7 (77%)

#### Theme 1: Acceptability of different techniques

The acceptability of different techniques varied between women. This is illustrated in Table 2 which summarises the contrasting choices of techniques that different women preferred. Variation in acceptability of different techniques appeared to be due to three key subthemes: *Level of invasiveness*, *Security in practitioner expertise* and *Baby safety*.

**Table 2:** Summary of women’s views on which technique(s) they preferred

Techniques	Justification for choice
Head down or the push technique	*… probably the ones where the pushing and the head down tilt one, feels a bit more natural, I would say …*. *cos it feels like you’re, for the way you described it, unless I have misunderstood it, it feels like me, as the mother who’s trying to give birth to my child, I still am trying to give birth to my child with some more assistance. Whereas, these feel completely, the power’s more out of my hands a little bit. Yeah.* (P1)
Tydeman tube	*… I prefer the tube because the doctor would be holding on to the bottom … and the air as well, will release a suction, so cos that will have a benefit over just using your hand because they’ll be able to get rid of the suction with the air … the hand you have better control …* (P2)
Fetal pillow	*I suppose this one [pillow] would be better than that [tube]. ...this pillow it seems, it looks erm, I don’t know about the right word, don’t look as hard as the tube … I think to be pushing on a baby’s soft skull. I think they’ve had enough sort of trauma down there already and the head’s getting squashed. And then to be coming from that way pushing them up … but maybe the pillow feels like a soft sort of ‘fabric’.* (P3)
Tydeman tube or the Fetal pillow	*Any of those techniques would be preferable to having hands or really physically pulling on a baby … I think sometimes you can feel like you are being manhandled I think and people can be a bit rough* (P4)
	*Um, possibly the pillow and the tube, maybe. I think the, I don’t know how to pronounce it, Patwardhan Method, is the furthest method away. That really seems like a very, very, within an emergency, an extra emergency kind of procedure to me.* (P5)
	*… if I had a choice in terms of how, if that technique was gonna be used, I’d rather something like this, it would be slightly more gentle … than the other, like the push technique* (P6)
Health professionals’ decision	*None of them seem particularly unacceptable or you know, there was nothing that I thought, oh, I really wouldn’t want to have that done to me … … I would put my hand in the health professional to be choosing the right implement … cos you don’t really have a choice anyway.* (P7)
Any technique	*I suppose I would be happy with whatever you had to use really. I suppose you trust the doctor to make the right decision don’t you and whatever you need to do to make sure the baby is safe really.* (P8)
	*I would go with anything. I’m quite trusting of medicine, and if something has to be done then that’s what has to be done, you know.* (P9)

##### Level of invasiveness

When weighing up the acceptability of one technique over another, women often talked about the extent to which an approach was invasive or intrusive to them or the baby. Women differed in what they perceived as intrusive, be it a clinician’s hand, instrument or a physical approach.

*.. you want the least intrusive thing that you can get hold of, really. And they don’t seem as bad as some of the alternatives … like, the head down tilt thing seems less invasive than some of the ones where you'd be using instruments, and that sort of thing.* (P5)

Another participant described a sense of safety of the doctor’s hand as it is viewed as something that has a high level of sensitivity and functionality compared with a tool or inanimate object.

*So erm yeah … , perhaps it’s safer to actually have somebody doing that, with their own hand, rather than a plastic implement* (P7)

Invasiveness was also understood in terms of potential risk of infection. This participant talks about the importance of hygiene.

*I suppose I prefer the tube or the pillow rather than the midwife hands I think … they just seem a bit more hygienic and a bit more cleaner* (P8)

##### Security in practitioner expertise

Some women talked about trusting the medical team to use the appropriate technique.

*I suppose I would be happy with whatever you had to use really. I suppose you trust the doctor to make the right decision don’t you and whatever you need to do to make sure the baby is safe really.* (P8)

A few women mentioned that the technique used was less important than ensuring women feel secure and reassured by the clinical team during the emergency situation.

*I think it’s, that it is really important in terms of making sure mum’s emotional wellbeing is you know at the forefront in terms of.. ‘cos you’ve got to perform this surgery, it’s huge surgery, you need to make sure that she feels secure in your care so that actually when she leaves there she’s like okay (laughs) well that’s happened* (P6)*.*

##### Baby safety

Lastly the extent to which a technique may damage or threaten the safety of their baby was mentioned by some women when considering acceptability.

*The other things … . Tocolysis, I’m not sure, I would probably put that as more, last resort if, obviously the main thing is to save the baby, so I would do anything if the baby was in trouble* (P2)

Another participant emphasised the importance of the baby arriving safely and less concerned about if it is damaged during the process.

*Even if it meant to deliver your baby we had to break the baby’s leg, it sounds horrific but I personally, would much rather that than not have a baby.* (P9)

#### Theme 2: informed choice in trials

Women’s views of a trial of different techniques produced the theme *Informed choice in trials* which had four subthemes of: *Timing of the invitation, Capacity to make an informed choice, Birth outcome* and *Importance of rapport*.

##### Timing of the invitation

Timing of an invitation to take part in a trial was important, as being offered information before birth would allow women time to understand and reflect on the project.

*I think it's quite a stressful time anyway, and there is quite a lot going on. … … if you are asked earlier on in the process, then you have got more time to sort of think about it properly, if that makes sense and … give a … sort of more informed right choice* (P8)

##### Capacity to make an informed choice

There was a consistent view from women that under critical conditions consenting to a trial would be challenging. Furthermore, in an emergency situation they might be more compliant and agree to anything, therein not being able to consider information thoughtfully.

*I appreciate all of that and I’d have been more than happy to be part of it, as I am now, but I just think you can’t ask people at those times. I just don’t know if they have full capacity even … I just wasn’t even thinking right …* (P3)

##### Birth outcome

Women described a willingness to take part in research once they were confident their baby was safe and well.

*After the baby’s nicely, safely delivered, so that would be, I’d of said yes to anything when my baby was here safely* (P4)

*After the baby has been … yeah that probably will be better, yeah because yeah you are sort of almost … you have gone through the process and you are relieved that everything is okay.* (P8)

##### Importance of rapport

Lastly, participants described the importance of being approached for research purposes by a clinician they knew; most women identified their midwife.

*I think maybe more by a midwife than anybody else … cos you have that, you have more of a relationship with your midwife than anybody else* (P4)

#### Theme 3: Birth education 

Women spontaneously reflected on their own birth experience and what would have been helpful in hindsight. The theme *Birth education* emerged from women reflecting on their experiences of having a second stage emergency CS and the need for education and knowledge before and after. This had two subthemes of *Antenatal education* and *Postpartum information.*

##### Antenatal education

Antenatal education and knowledge were seen as an opportunity to have some control over the impact of birth events as opposed to being blind to potential adverse events.

*Going into a situation you know nothing about it takes away a lot of your control I think … you wouldn’t do this for any other surgery, you wouldn’t approach any other situation without the full picture, but you present women who are pregnant with this almost glorified text book* (P4)

Participants reflected on whether it’s important for women to be informed about all types of birth outcomes, not just positive ‘glorified’ births. Women raised the importance of reframing narratives around CS at antenatal classes as like any other type of birth in order to minimise a sense of failure among women who have a CS.

*So actually I think there probably is a lot more education that could be available so people … don’t feel this is a weird way to give birth, but it’s still a way to give birth* (P4)

##### Postpartum information

Similarly, women raised the importance of information postpartum to understand events during birth. Women discussed the value of processing the events of birth afterwards. They discussed the value of knowledge in validating their experiences and alleviating the negative emotional impact.

*… .Because I had to stay in hospital for five days afterwards. Um, and he just came back and sort of said, “Do you know what's happened to you?” (Laughs). And I said (High voice), “Ooh no, I don’t think I do.” (Laughs). I was very emotional. And then he explained it all to me, and that actually made me feel 1,000 times better, just him taking five minutes just to explain that to me* (P1)

## Discussion

### Main findings

This study aimed to determine the acceptability of different techniques for managing impacted fetal head at CS; and the feasibility and acceptability to women of conducting an RCT of those techniques. Women varied in which technique they thought was most acceptable. Women’s trust in medical expertise and prioritising the safety of the baby were important moderators of acceptability of techniques. Greater consensus was found on factors important to consider in a future RCT. These included timing of consent, capacity to consent in emergency situations, the importance of birth outcomes, and good rapport with the consenting clinicians. Women also reflected on antenatal education and postpartum information being important when complications like impacted fetal head arise.

### Strengths and limitations

This research is the first to examine the acceptability to women of different techniques to manage impacted fetal head and their views on a possible RCT to evaluate techniques. Limitations of the study are that the sample was small and included a high proportion of women educated to degree level or above, most women were working and, incidentally, a third of these women worked in healthcare. All women were White British. The sample is therefore not representative and the results not generalisable. The consistency of our findings with previous literature suggests some elements may be generalisable but further research is needed to look at the acceptability of techniques to manage impacted fetal head in women from other socio-economic and ethnic groups. As women were interviewed up to 2 years after their CS it is possible there is an element of recall bias in their responses, although research suggests women have accurate recall of birth events 4–6 years postpartum [11].

### Interpretation

This study did not identify one technique that would be acceptable to all women. Preferred techniques included: fetal pillow, Tydeman tube, head down tilt and push technique. No woman chose reverse breech extraction, tocolysis or Padwardhan technique. These views are consistent with previous research: a meta-review of 43 reviews of acceptability of healthcare interventions highlighted that acceptability is a multifaceted construct which involves individual’s thoughts, feelings and behaviour, and can be individually and/or socially acceptable to patients or health professionals [12]. Acceptability can be prospective based on anticipated responses, or retrospective, based on experience [12]. The acceptability of an intervention is also likely to vary according to the content, context and quality of care [12]. This is consistent with our findings that trust in medical expertise and prioritising the safety of the baby in emergency situations influenced acceptability of techniques.

This has a number of implications. First, our participants reported acceptability in relation to anticipated responses, not known past experience. However, participants had all experienced second stage CS so the interviews sometimes prompted the realisation that they may have experienced impacted fetal head and one of these techniques may have been used without them knowing which one. Results in relation to acceptability are therefore not based on known experience of different techniques, but were conducted with women who had been in similar, or closely related, circumstances. Second, the importance of context and quality of care is evident across all themes, especially in subthemes of *Baby safety*, *Birth outcome*, *Security in practitioner expertise* and *Importance of rapport*. Similarly, it could be argued that the theme of *Education* is predominantly about context (i.e. women’s knowledge) and care (i.e. antenatal and postnatal education and information).

Findings about acceptability of a trial are consistent with literature on trials of procedures in emergency situations. Findings about the importance of informed choice are consistent with guidelines that emphasise informed consent for research must include capacity, disclosure, understanding, voluntariness and permission [13]. The difficulty obtaining informed consent in emergency or critical situations has been widely debated. Although women in this study wanted information beforehand in order to consider it, the advantages and disadvantages of this are recognised in guidelines for obstetric research [14]. In some circumstances informing women in advance might create unnecessary anxiety, particularly if the obstetric complication is rare [14]. On the other hand, trying to obtain consent during critical situations may delay life-saving treatment and if women are under stress they may lack capacity to give fully informed consent [15].

Different proposals for gaining consent in emergency circumstances have been proposed, including deferred consent (requested after the emergency and procedure) [16], or a two-stage process of verbal consent during the emergency followed by written consent after the event [17]. A qualitative study of women’s experiences of consent during or after birth (i.e. deferred consent) found that women’s experiences of trial participation were the same regardless of the way in which they gave consent. The authors concluded clinicians’ understanding of a woman’s individual situation and experience is paramount in negotiating consent during obstetric emergencies [16].

Thus, context and quality of care is important in both acceptability of interventions to women as well as consent procedures. This is consistent with our findings that women wanted to feel *security in practitioner expertise* and emphasised the *importance of rapport* with the clinician who invited them to take part in a trial. Similarly, safety of the baby was mentioned in relation to acceptability of techniques to manage impacted fetal head (*baby safety*) and in relation to obtaining consent (*birth outcomes*).

The theme of *Birth education and information* emerged spontaneously. Women thought antenatal education could have prepared them better for their birth experiences, and that postpartum information was helpful. It is increasingly recognised that some women find birth traumatic and complications such as emergency CS are a risk factor [18]. Although it should be noted that women’s subjective birth experience is more strongly associated with traumatic stress reactions to birth [19].

## Conclusion

Results offer insights for health professionals working with emergency obstetric procedures such as impacted fetal head. Findings highlight the individual variability in the acceptability of techniques for managing impacted fetal head to different women, as well as the importance of context and quality of care. In approaching women to take part in a future trial, flexibility of approaches is required. Ideally women would have time to consider and consent before the critical situation arises but, if not, full written consent should not be sought in critical or emergency situations when women have limited capacity. Ideally, women should be approached by a healthcare professional known to them.

Further research is needed to consider acceptability in different groups of women, such as those from different socio-economic and ethnic backgrounds, as well as women who have experienced impacted fetal head and one of the techniques. Acceptability of techniques to healthcare professionals expected to use these techniques in a future trial is also important to examine in conjunction with women’s views.

## Supplementary Information

**Additional file 1.**

## Data Availability

The dataset supporting the conclusions of this article is available from the corresponding author on request.

## Abbreviations

CS: Caesarean Section
RCT: Randomized Controlled Trial
IMD: Index of Multiple Deprivation Decile
