# Supplementary material for: The acceptability to women of techniques for managing an impacted fetal head at caesarean section and of randomised trials evaluating those techniques: a qualitative study
Source: BMC Pregnancy Childbirth. 2021 Feb 2;21:103. doi: 10.1186/s12884-021-03577-z (PMC7852117; doi:10.1186/s12884-021-03577-z)
Supplement: Supplementary file 1 — Additional file 1. [file 12884_2021_3577_MOESM1_ESM.docx]

**APPENDIX S1**

Presentation script of techniques for managing an impacted fetal head used in semi-structured interviews. Used in conjunction with PowerPoint illustrations and physical props where applicable (Tydeman tube and Fetal pillow).

**Pelvis Introduction**

- This is your bony pelvis
- It’s like a funnel
- The muscles of pelvic floor form a sling that is seated at the bottom of this
- At end of labour baby’s head can be very wedged in pelvis
- At caesarean, when birth is straight forward, put hand in under baby’s head, lift head out
- When there is an impacted fetal head it can be very tight, vacuum between head and pelvic floor, head wedged.
- Always aiming to deliver the presenting part - the part of the baby that is closest to your incision
- When the baby gets stuck/impacted for different reasons baby might be in wrong position e.g. looking up at the sky rather than to one side, woman contracts but labour not progressing, head gets more and more wedged in pelvis.
- Commonly – tried to deliver with instrument, use forceps or suction cup, so because pulled- head gets even more stuck

**Fetal Pillow**

- Firm but flexible
- Balloon you can inflate and has tubing
- Connect syringe with warm saline and fill pillow up
- Put through vagina, onto babies’ head
- As you fill with saline it pushes the baby’s head up
- Sometimes you can use if you think you will encounter difficulty or when you actually encounter difficulty
- Most units use when a problem is encountered

**Tydeman Tube**

- Silicon tube, quite bendy
- Has a hollow centre
- Idea is that it is placed in vagina
- Fits perfectly on babies’ head (at an angle so that when elevation occurs the baby’s head is flexed)
- Elevates and breaks the vacuum because introduces air through hollow tube
- You get an assistant to place tube, put on babies’ head and push upwards
- Helps break vacuum and push head
- Has advantage of hollow tube to help with vacuum
- Making you put pressure on the right place

**Push Technique/Pressure from below**

- Using flats of fingers to flex and elevate head.
- Performed by a midwife or colleague
- Your assistant is performing the manoeuvre that you usually do at caesarean to achieve delivery but can’t in this situation

**Reverse Breech Extraction**

- At Caesarean section, can’t get under baby’s head. Tried all techniques.
- Very infrequent. In UK it is last resort.
- Put hand in through abdominal incision, go right up to the top of the womb and grab the baby’s leg.
- Pull baby feet first, through the incision
- Babies feet are right at top of the womb

**Tocolysis**

- Give mum a medicine that will relax the womb
- When put hand in to get head, the womb contracts to the stimulus of you putting your hand in
- Medication to relax the womb – gives you more room to manoeuvre
- Spray of medicine under the tongue or an injection with needle under the skin
- They work in 2 minutes, relax womb
- Negative sides:
  - May help deliver but….
  - It increases the risk of bleeding
  - Don’t want to give at time of caesarean, normally give medication to make womb contract so don’t bleed too much
  - Side effects of the medication one commonly used medication can cause palpitations
  - Another commonly used medication for this purpose can cause headaches

**Head Down Tilt**

- Ask anaesthetist to tilt head of operating table down, gravity helps to assist with delivery
- Negatives: Not pleasant for the woman to be placed head down, in some women (high BMI) anaesthetist may not wish to place head down

**Patwardhan Method**

- Described in the literature mainly in the developing world
- Can’t get baby’s head
- So pull on baby’s shoulder
- If head very impacted and pull too hard – can get injury to nerves in the neck
- Never taught in the UK
